# Reassessing the Prognostic Value of Lymph Node Metastasis in Deficient Mismatch Repair Colorectal Cancer

**DOI:** 10.3390/curroncol32050254

**Published:** 2025-04-27

**Authors:** Zilan Ye, Dakui Luo, Fan Chen, Jiayu Chen, Zezhi Shan, Junyong Weng, Yu Zhang, Qingguo Li, Xinxiang Li

**Affiliations:** 1 Department of Colorectal Surgery, Fudan University Shanghai Cancer Center, Shanghai 200032, China; yzlsibyl@163.com (Z.Y.); dkluo17@fudan.edu.cn (D.L.); 19301050220@fudan.edu.cn (F.C.); chenjyhhh@163.com (J.C.); 18111230016@fudan.edu.cn (Z.S.); jerome6694@163.com (J.W.); yuzhang24@m.fudan.edu.cn (Y.Z.); 2 Department of Oncology, Shanghai Medical College, Fudan University, Shanghai 200031, China

**Keywords:** log odds of positive lymph nodes, deficient mismatch repair, colorectal cancer, lymph node metastasis, prognostic factor

## Abstract

Background: In non-metastatic deficient mismatch repair (dMMR) colorectal cancer (CRC), traditional prognostic factors, such as pN staging, often fail to distinguish patient outcomes effectively. Methods: This retrospective study included a cohort of 792 dMMR CRC patients who underwent surgical treatment without neoadjuvant chemoradiotherapy or immunotherapy. Traditional prognostic factors were compared with lymph node-based models (NLN, LNR, LOODS) for their ability to predict overall survival (OS) and disease-free survival (DFS). Results: The study demonstrated that traditional factors, such as histologic type, differentiation, and vascular invasion, had limited predictive value in dMMR CRC. Furthermore, the pN stage failed to effectively distinguish between pN1 and pN2 for both OS (*p =* 0.219) and DFS (*p =* 0.095). Conversely, LOODS demonstrated superior performance over traditional pN staging in predicting both OS and DFS (*p* < 0.001). A prognostic model combining LOODS with age exhibited superior predictive performance compared with the traditional TN staging system. Conclusions: LOODS was identified as a more effective independent prognostic factor compared with traditional pN staging, enabling more precise stratification of pN+ patients in non-metastatic dMMR CRC, highlighting its potential utility in guiding postoperative treatment and optimizing therapeutic strategies.

## 1. Introduction

Colorectal cancer (CRC) ranks as the third most common cancer globally and represents the second leading cause of cancer-related deaths, highlighting a major global health challenge [1]. CRC encompasses a heterogeneous group of diseases with distinct subtypes, each characterized by specific molecular alterations. These subtypes exhibit significant variability in progression, metastasis, treatment response, and prognosis. The deficient mismatch repair (dMMR)/microsatellite instability (MSI) subtype of CRC arises from mutations or alterations in mismatch repair (MMR) genes and accounts for approximately 15% of stages I–III CRC [2].

Clinically, dMMR CRC often presents at a younger age, predominantly involves the right-sided colon, and occurs more frequently in early-stage cases, generally indicating a favorable prognosis, particularly in stages II and III [3,4,5,6]. dMMR CRC exhibits strong responsiveness to immunotherapy, which can be attributed to its high levels of immune cell infiltration and robust tumor immune response [7,8]. However, substantial evidence indicates that chemotherapy, particularly regimens involving 5-FU, has reduced efficacy in dMMR CRC patients compared with those with proficient MMR (pMMR) [9,10,11,12].

Given the heterogeneity of dMMR/MSI CRC, traditional prognostic factors are often insufficient to reliably stratify patients for clinical decision-making in non-metastatic cases. Notably, several established CRC guidelines exhibit considerable inconsistencies in treatment recommendations for dMMR/MSI CRC, particularly for stage II patients with high-risk factors [13,14,15,16]. Therefore, in the absence of robust evidence supporting immunotherapy in non-metastatic dMMR/MSI CRC, the development of an effective clinical prognostic model is essential for identifying patients with poorer outcomes.

In non-metastatic CRC, N staging serves as a critical prognostic factor. However, it primarily depends on the enumeration of positive lymph nodes without accounting for the total number of lymph nodes examined. This approach is often insufficient for a thorough assessment of lymph node metastasis, particularly when the total lymph node count varies significantly among patients. Furthermore, the prognostic value of N staging significantly declines when fewer than 12 total lymph nodes are analyzed [17]. This limitation may result in under-staging, thereby affecting treatment decisions and overall prognosis. Consequently, incorporating additional factors beyond the simple enumeration of positive lymph nodes could enhance the prognostic reliability of N staging in CRC.

Several studies have demonstrated that dMMR/MSI CRC is linked to an increased lymph node yield and reduced lymph node metastasis, culminating in a higher count of negative lymph nodes [18,19,20,21]. Consequently, the integration of negative lymph nodes into enhanced staging models is essential for dMMR/MSI CRC, a subtype characterized by a lack of robust prognostic factors. Lymph node-based prognostic factors, including the number of negative lymph nodes (NLNs), lymph node ratio (LNR), and log odds of positive lymph nodes (LODDS), have demonstrated efficacy in predicting outcomes in other cancers [22,23,24]. The LODDS model incorporates both the total lymph node yield and the positive lymph node rate, which provide more information in the context of dMMR CRC.

This study aimed to reassess the prognostic value of lymph node metastasis in dMMR CRC by comparing the prognostic significance of three lymph node-based models, and identifying additional prognostic factors that may assist in risk stratification and clinical decision-making.

## 2. Materials and Methods

### 2.1. Study Population

This study retrospective analyzed 1001 dMMR CRC patients who underwent radical surgery at a university teaching cancer center between January 2014 and December 2019, ultimately including 792 cases. All patients underwent postoperative pathological confirmation. Exclusion criteria included stage IV disease, prior neoadjuvant chemoradiotherapy, and incomplete pathological or follow-up data. Immunotherapy was not administered to any patients. There were 130 cases of rectal cancer, accounting for 16.4%, 150 cases of left-sided colon cancer, making up 18.9%, and 512 cases of right-sided colon cancer, which is 64.6%.

The data extracted from the database encompassed immunohistochemical results, age at diagnosis, sex, histological type, tumor differentiation, the number of total lymph nodes (TLNs) examined, the number of positive lymph nodes (PLNs) examined, pathological T (pT) and N (pN) staging, perineural invasion, vascular invasion, surgical details, and survival outcomes. The study protocol was approved by the ethical committee and institutional review board of the university teaching cancer center.

### 2.2. Postoperative Follow-Up

The postoperative follow-up protocol comprised clinical examinations, tumor marker evaluations, chest, abdomen, and pelvic CT scans, as well as colonoscopies. Survival data were obtained from medical record reviews, telephone follow-ups, and death registry entries. The primary survival outcomes evaluated included overall survival (OS) and disease-free survival (DFS). OS was defined as the time from the initiation of treatment to death from any cause or the last follow-up. DFS was defined as the time from the initiation of treatment to either local or distant recurrence, death from any cause, or the last follow-up.

### 2.3. Deficient Mismatch Repair

Tumor MMR status was assessed through immunohistochemical staining of four MMR proteins: MLH1, MSH2, MSH6, and PMS2. Tumors with negative expression of one or more of these proteins were categorized as dMMR. All tumors analyzed in this study were verified to exhibit dMMR status.

### 2.4. Lymph Node-Based Prognostic Factors

According to the 8th edition of the American Joint Committee on Cancer (AJCC) staging system, patients are categorized into N0, N1, and N2 subgroups according to N staging [25]. The NLN count is determined by subtracting the PLN count from the TLN count. The LNR represents the ratio of PLNs to TLNs, while the LODDS is calculated using the formula: log((PLN + 0.5)/(NLN + 0.5)). The optimal cutoff values for NLN, LNR, and LODDS were established using X-Tile software (version 3.6.1, https://medicine.yale.edu/lab/rimm/research/software/ accessed on 6 November 2024) to classify these variables into three distinct groups [26,27,28,29,30]. Using statistical tests (e.g., log-rank for survival) generates a triangular heatmap where color intensity (green/red for positive/negative associations) reflects the strength of correlation with clinical outcomes. The highest chi-square (χ^2^) value selects the optimal cut-off, validated internally via training-validation splits and externally with statistical software. Harvesting at least 12 TLNs during surgery is considered standard practice and serves as a benchmark for evaluating TLN counts. To compare the predictive performance of prognostic models combining LODDS and age with conventional pT and pN staging, three additional models were analyzed:

Model 1: pN + pT (pT1/pT2 vs. pT3/pT4).

Model 2: LODDS + EOCRC (EOCRC vs. LOCRC).

Model 3: LODDS + critical age (<77 years vs. ≥77 years).

The critical age was established based on the optimal value identified through X-Tile software analysis.

### 2.5. Statistical Analysis

All statistical analyses were performed using SPSS (version 27.0) and R software (version 4.3.1). Nominal variables were analyzed using the chi-squared test, while ordered multicategorical variables were analyzed using the Mann–Whitney U test. OS and DFS were assessed and compared through the Kaplan–Meier method. Independent prognostic factors were identified through univariate and multivariate Cox regression analyses. The predictive performance of various models was evaluated through the receiver operating characteristic curve (ROC), time-dependent ROC (TimeROC), and concordance index (C-index). The R packages ‘survivalROC’, ‘riskRegression’, and ‘pec’ were employed for analysis and visualization. Statistical significance was defined as a *p*-value of less than 0.05.

## 3. Results

### 3.1. Demographic and Clinicopathological Characteristics

The study comprised 792 patients meeting the inclusion criteria. The detailed process of patient inclusion and exclusion is shown in Figure S1. The cohort included 420 (53.0%) males and 372 (47.0%) females. Of these patients, 216 (27.3%) were diagnosed with early-onset CRC (EOCRC), whereas 576 (72.7%) were diagnosed with late-onset CRC (LOCRC). The distribution of cancer stages was as follows: 119 (15.0%) patients were at stage I, (57.7%) 257 at stage II, and (27.3%) 416 at stage III.

Detailed baseline characteristics of the patients are summarized in Table S1.

### 3.2. Lymph Node-Based Prognostic Models

**Figure 1:**
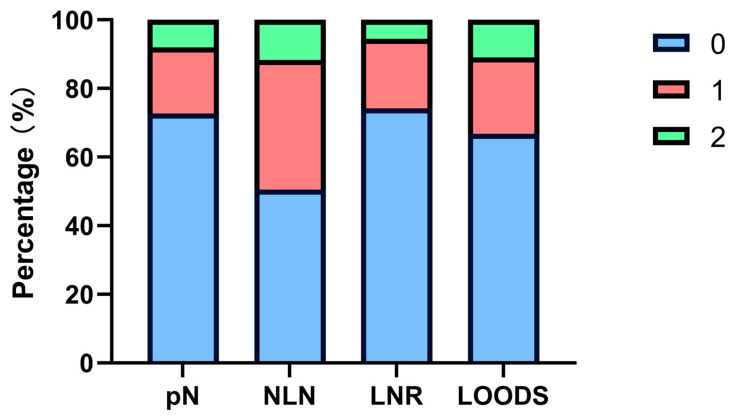
Patient stratification proportions of four lymph node-based prognostic models.

The mean TLN count was 21.93, while the mean PLN count was 0.88. It is noteworthy that only 30 patients (3.8%) had fewer than 12 TLNs retrieved during surgery. The variables NLN, LNR, and LODDS were classified into three subgroups each, according to their optimal cut-off values (Figure 1, Figure S2):

NLN was grouped into NLN0 (NLN ≥ 20), NLN1 (13 ≤ NLN < 20), and NLN2 (NLN < 13).

LNR was grouped into LNR0 (LNR = 0), LNR1 (0 < LNR < 0.26), and LNR2 (LNR ≥ 0.26).

LODDS was grouped into LODDS0 (LODDS < −1.440), LODDS1 (−1.440 ≤ LODDS < −0.764), and LODDS2 (LODDS ≥ −0.764).

### 3.3. Survival Analysis of Traditional Prognostic Factors in dMMR CRC

The median follow-up duration for this cohort was 40.3 months. Both univariate and multivariate Cox regression analyses were performed to assess the prognostic relevance of traditional prognostic factors in patients with dMMR. After adjustment for established confounders, pN stage (pN1 vs. pN0, HR: 5.547, 95% CI: 2.722–11.303, *p <* 0.001; pN2 vs. pN0, HR: 7.728, 95% CI: 3.348–17.839, *p <* 0.001) and age (EOCRC vs. LOCRC, HR: 0.394, 95% CI: 0.185–0.839, *p =* 0.016) emerged as independent prognostic factors for OS, while only pN stage (pN1 vs. pN0, HR: 4.147, 95% CI: 2.246–7.657, *p <* 0.001; pN2 vs. pN0, HR: 6.243, 95% CI: 3.021–12.902, *p <* 0.001) remained significant for DFS. Notably, other commonly utilized prognostic factors, including histologic type, differentiation, vascular invasion, and perineural invasion, failed to demonstrate statistical significance in the dMMR CRC cohort (Table 1, Table 2).

### 3.4. Survival Analysis of Lymph Node-Based Prognostic Models

**Figure 2:**
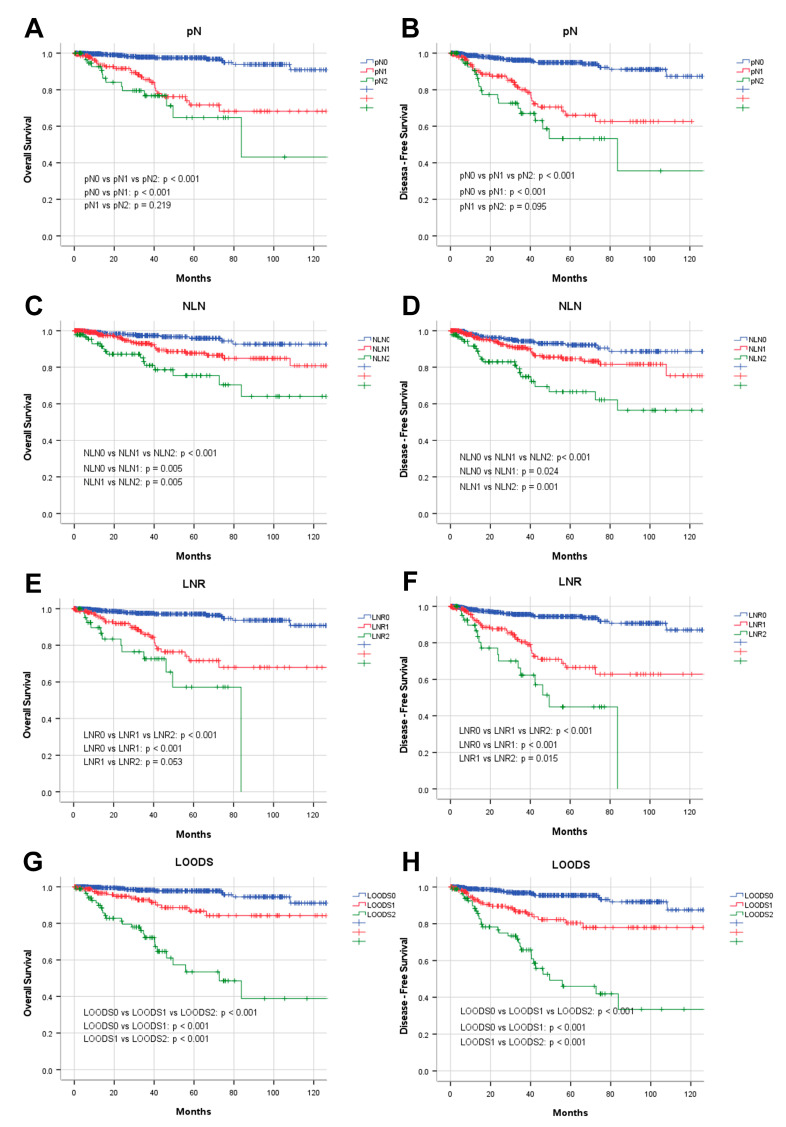
Prognostic analysis of four lymph node-based prognostic models. Kaplan–Meier analysis of OS in patients stratified by (**A**) pN, (**C**) NLN, (**E**) LNR, and (**G**) LOODS; Kaplan–Meier analysis of DFS in patients stratified by (**B**) pN, (**D**) NLN, (**F**) LNR, and (**H**) LOODS.

Four lymph node-based prognostic models were used to stratify patients, and their stratification effects were compared. Significant differences in OS and DFS were observed among patients categorized using the four prognostic models (*p <* 0.001). However, in patients with pN+ disease, the pN stage failed to effectively distinguish between pN1 and pN2 for both OS (*p =* 0.219) and DFS (*p =* 0.095). Of the evaluated models, the LODDS exhibited the highest discriminatory capacity. The five-year OS rates for patients categorized as LODDS0, LODDS1, and LODDS2 were 97.7%, 86.7%, and 53.4%, respectively (*p <* 0.001). The three-year DFS rates for these groups were 96.8%, 86.5%, and 65.8%, respectively (*p <* 0.001; Figure 2).

In subsequent analyses, pN staging was substituted with alternative prognostic models in a multivariable Cox regression to evaluate their prognostic significance. The findings verified that NLN, LNR, and LODDS function as independent prognostic indicators for both OS and DFS (Table 3).

**Figure 3:**
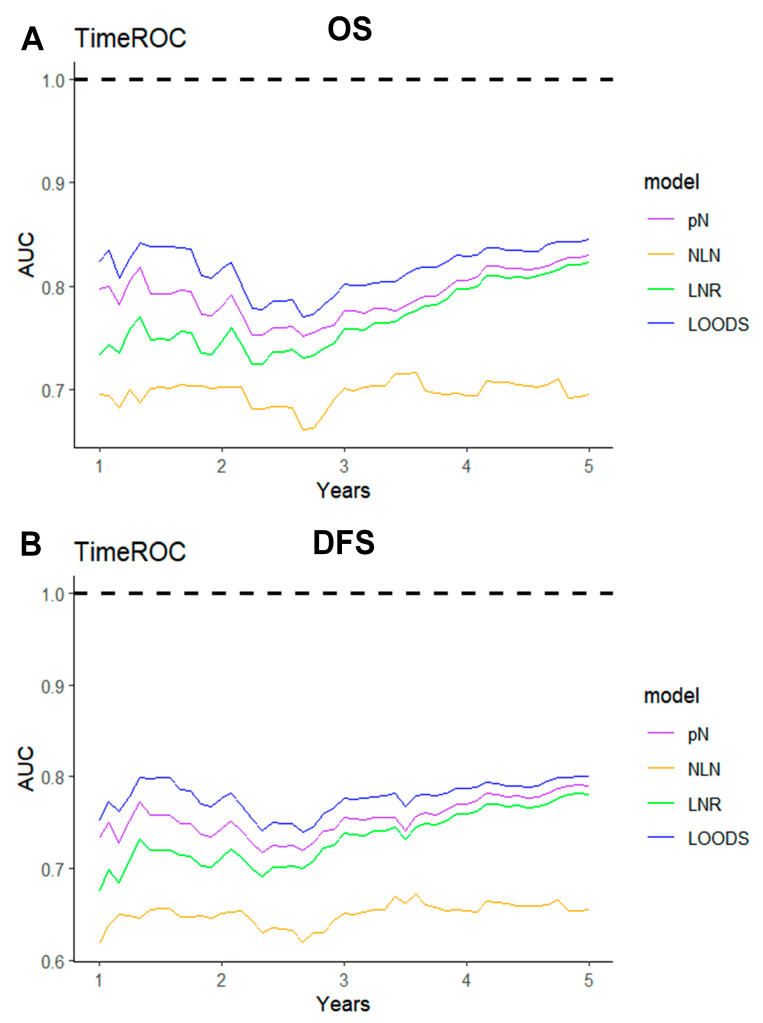
TimeROC analysis comparing the predictive performance of four lymph node-based prognostic models. TimeROC curves for (**A**) OS and (**B**) DFS of four models.

**Figure 4:**
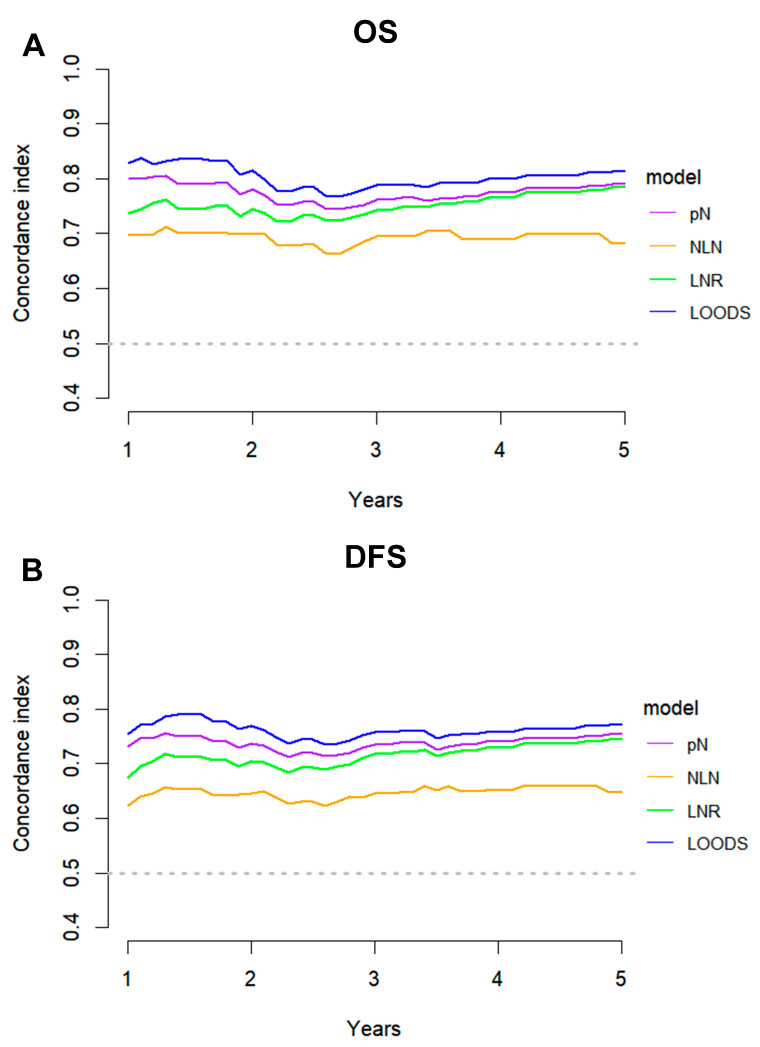
C-index analysis comparing the predictive performance of four lymph node-based prognostic models. C-index curves for (**A**) OS and (**B**) DFS of four models.

The ROC method was employed to evaluate the predictive performance of four lymph node-based prognostic models for both OS and DFS. The area under the curve (AUC), a measure of the geometric area beneath the ROC curve, was used to quantify the predictive performance at 1-year, 3-year, and 5-year intervals. Among these models, the LODDS exhibited superior predictive performance, achieving a 5-year OS AUC of 0.831 and a 3-year DFS AUC of 0.768 (Figure S3). The TimeROC curve further substantiated that LODDS consistently surpassed the predictive performance of other models across all intervals (1 to 5 years) for both OS and DFS. Conversely, the NLN count demonstrated the weakest predictive performance (Figure 3). This observation was further corroborated by the C-index analysis (Figure 4).

**Figure 5:**
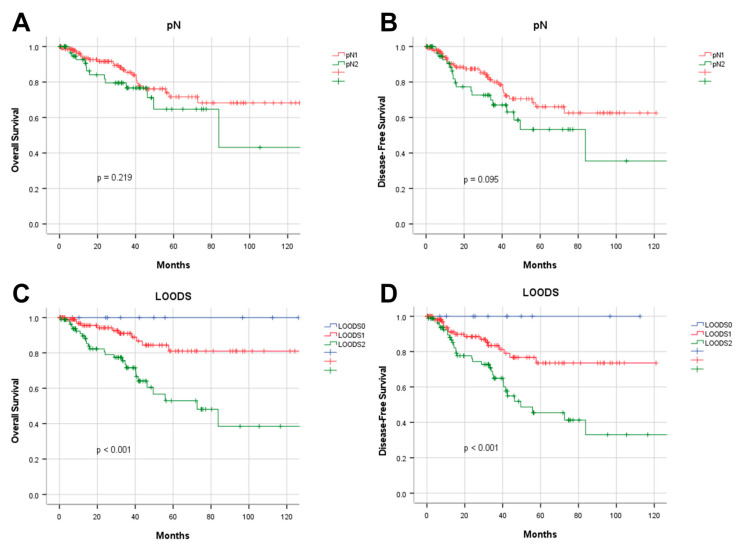
Prognostic analysis of pN and LOODS staging in pN+ patients. Kaplan–Meier analysis of overall survival in pN+ patients stratified by (**A**) pN and (**C**) LOODS; Kaplan–Meier analysis of disease-free survival in pN+ patients stratified by (**B**) pN and (**D**) LOODS.

As previously noted, pN staging demonstrated limited discriminatory power in pN+ patients as it did not incorporate the total number of lymph nodes (TLNs). To address this limitation, the LODDS staging system was applied, demonstrating superior stratification ability for both OS and DFS in pN+ patients (*p <* 0.001) (Figure 5). Notably, within the pN+ group, LODDS identified a distinct subgroup with a favorable prognosis, categorized as LODDS0.

### 3.5. Predictive Performance of the Combined LODDS and Age Prognostic Model

**Figure 6:**
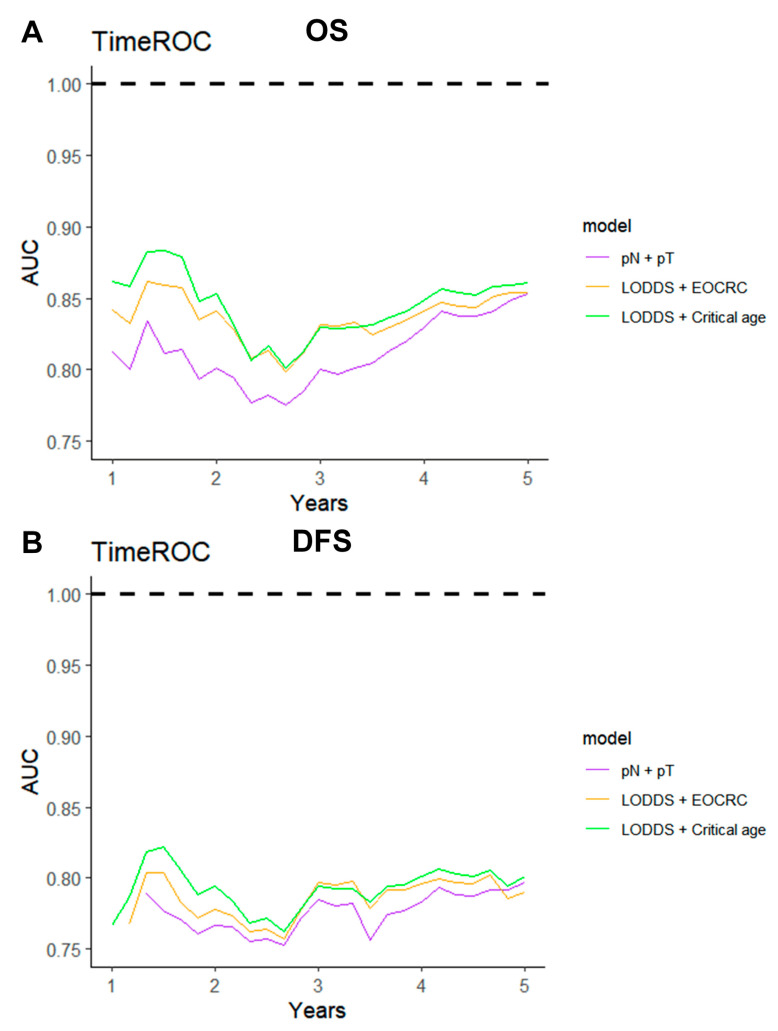
TimeROC analysis comparing the predictive performance of three combined models. TimeROC curves for (**A**) OS and (**B**) DFS of three combined models.

**Figure 7:**
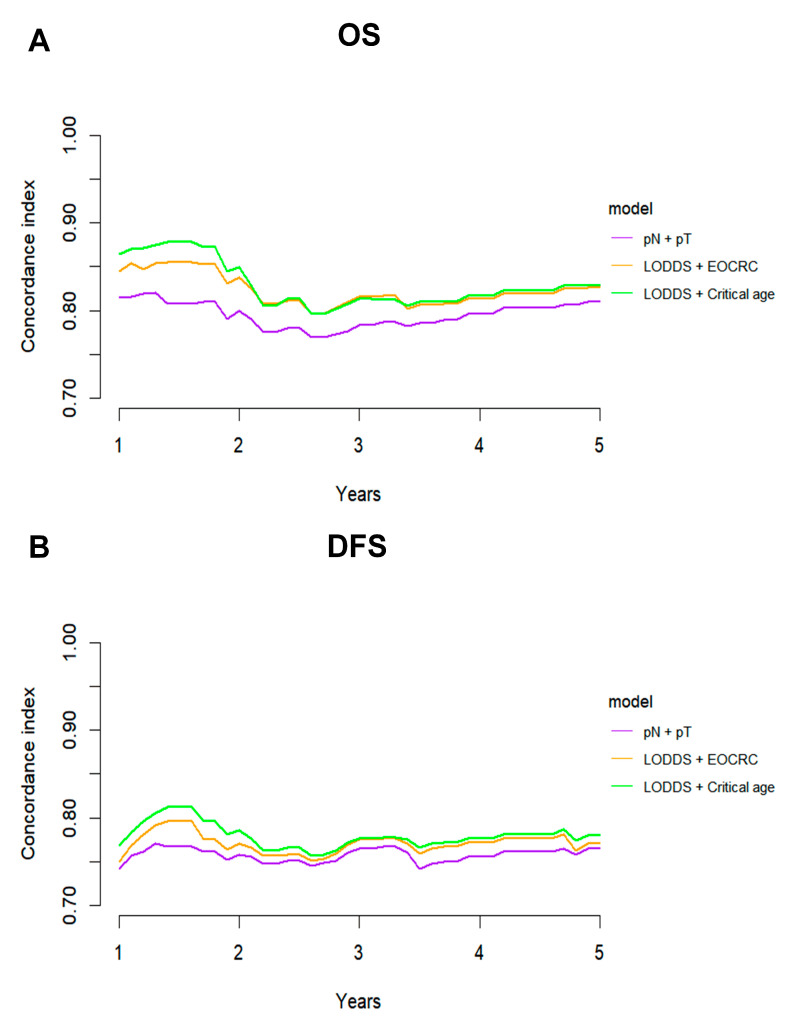
C-index analysis comparing the predictive performance of three combined models. C-index curves for (**A**) OS and (**B**) DFS of three combined models.

Given the diminished prognostic significance of traditional factors in patients with dMMR CRC, a combined model integrating the LODDS and age was developed and compared with the conventional TNM staging system. The predictive performance of the LODDS and age model was evaluated for both OS and DFS. Analyses employing TimeROC and C-index revealed that the LODDS and age model consistently surpassed the traditional TNM model, regardless of stratification by EOCRC vs. LOCRC or optimal cutoff values (Figure 6, Figure 7).

## 4. Discussion

Consensus on the remarkable efficacy of immunotherapy in treating metastatic CRC with dMMR/MSI has been firmly established, supported by multiple studies [31,32,33,34,35,36,37,38,39]. For non-metastatic dMMR CRC, numerous studies have demonstrated the feasibility and safety of immunotherapy in both adjuvant and neoadjuvant settings [40,41,42,43]. The latest NICHE-2 trial administered neoadjuvant nivolumab and ipilimumab to 111 non-metastatic dMMR patients, achieving a pathological response in 109 patients (98%), significant pathological responses in 105 patients (95%), and complete pathological remission in 75 patients (68%). The trial reported a three-year DFS rate of 100% [44]. Although immunotherapy has demonstrated strong therapeutic efficacy and clinical value in non-metastatic dMMR CRC, chemotherapy continues to be the standard treatment in current clinical practice. The effectiveness of chemotherapy, whether fluoropyrimidine- or oxaliplatin-based, in treating non-metastatic dMMR CRC remains a subject of debate [45]. One primary factor contributing to this debate, aside from the inherent heterogeneity of the studies, is the distinctive biology of dMMR CRC. Notably, dMMR CRC exhibits inherent resistance to chemotherapy. Moreover, traditional prognostic factors for CRC often fail to effectively stratify dMMR CRC patients, thereby hindering the accurate identification of patients most likely to benefit from chemotherapy.

Ongoing disagreement exists among widely recognized clinical guidelines concerning the appropriate selection of dMMR CRC patients for chemotherapy. The National Comprehensive Cancer Network (NCCN) and the Chinese Society of Clinical Oncology (CSCO) recommend observation and follow-up for high-risk stage II dMMR/MSI CRC, particularly in cases involving T4b tumors [14,16].

Conversely, the European Society for Medical Oncology (ESMO) guidelines recommend routine postoperative adjuvant chemotherapy for high-risk stage II dMMR/MSI CRC patients. These patients are identified based on criteria such as pT4 status, fewer than 12 lymph nodes examined, or multiple additional risk factors (e.g., lymphatic, perineural, or vascular invasion; histologic grade 3; tumor obstruction; tumor budding; or preoperative CEA > 5 ng/mL) [15]. This disparity arises primarily because high-risk factors in pMMR CRC are less predictive of poor outcomes in dMMR CRC. Therefore, it is crucial to investigate the prognostic significance of diverse high-risk factors in dMMR patients and to design more tailored prognostic models to guide effective treatment decisions effectively.

We conducted a prognostic analysis of 792 dMMR CRC patients, and the results revealed that many traditional risk factors exhibit significantly reduced prognostic value in dMMR CRC. However, pN staging and age remain significant prognostic factors [46,47,48,49,50,51]. DMMR CRC patients are typically characterized by more TLNs examined and fewer PLNs examined, resulting in a greater number of NLNs compared with pMMR CRC patients [20,52]. In contrast to pN staging, NLN, LNR, and LOODS incorporate negative lymph nodes into their calculations. It was observed that LOODS demonstrates stronger prognostic significance in dMMR patients and could potentially replace traditional pN staging. The prognostic value of LOODS in CRC has been evaluated across several studies, including in non-metastatic, metastatic, and neoadjuvant treatment patients [17,53,54,55]. Although these studies affirmed the potential and superiority of LOODS as a staging tool, their findings were constrained by small sample sizes. Moreover, as traditional prognostic factors are well-established and predictive, replacing pN staging with LOODS may seem unnecessary in general.

Additionally, LOODS could complement pN staging rather than serving as its alternative. It was observed that while pN staging remained an important independent prognostic factor, its ability to differentiate prognosis among pN+ dMMR patients was reduced. LOODS further enhanced prognostic accuracy by offering additional stratification when integrated with pN staging. Notably, LOODS remained a significant prognostic marker for pN+ patients. It effectively identified pN+ LOODS0 patients whose prognosis closely resembled that of pN0 patients. Postoperative adjuvant chemotherapy is currently recommended for stage III pN+ LOODS0 patients, according to clinical guidelines. However, the integration of LOODS for further stratification could exempt these patients from chemotherapy, thereby alleviating unnecessary health burdens.

In our study, NLN was identified as the least effective lymph node-based prognostic model. However, a similar analysis by Dai et al. concluded that NLN was the strongest prognostic factor among pN staging, NLN, and LNR in dMMR CRC patients [56]. This discrepancy could be attributed to several factors, including our larger sample size. NLN was categorized into four subgroups based on quartiles by Dai et al., whereas pN staging and LNR were divided into only two or three subgroups. Different grouping methods could influence the comparison of predictive performance. Relying solely on quartiles to divide the population into four groups may not be clinically representative, potentially compromising the reliability of the conclusions drawn. Additionally, studies have shown that the lymph node yield in dMMR/MSI CRC surgery tends to be higher [18,19,20]. However, 16.3% of dMMR patients in Dai et al.’s study had fewer than 12 lymph nodes harvested, compared with only 3.8% in our study, which more closely aligns with the standards for surgical lymph node clearance. Moreover, when multiple lymph node-based factors are included in a multivariate Cox model, multicollinearity is unavoidable. Thus, LOODS was considered a superior prognostic factor compared with pN and NLN in dMMR patients.

Our study revealed that age is an important prognostic factor in dMMR CRC. Given the diminished value of traditional prognostic factors in dMMR CRC, we tested combined models of LOODS and age, which outperformed the widely used clinical TN staging. Previous studies demonstrated that EOCRC are more likely to exhibit dMMR/MSI, which is frequently associated with Lynch syndrome [57,58,59,60]. Therefore, EOCRC may significantly influence dMMR CRC patients by impacting genetic syndrome prevalence and driving the occurrence of dMMR/MSI. Despite being the most common and easily accessible prognostic factor in clinical practice, age is frequently overlooked in prognostic studies. Its prognostic value in dMMR patients warrants further exploration.

First, as a university teaching cancer center, patients with more advanced cancers from diverse regions are more likely to be admitted. Second, the diagnosis of dMMR/MSI CRC relied solely on immunohistochemistry, without polymerase chain reaction validation, even though both methods are generally considered equally accurate [61]. Finally, while immunotherapy and neoadjuvant therapy were confirmed to have not been administered to any patients, detailed data regarding postoperative chemotherapy were unavailable.

Recent evidence underscores the dual role of ctDNA [62] in advanced CRC as both a prognostic marker and early predictor of treatment response, with its ability to dynamically monitor tumor evolution and resistance mutations under therapy, thereby refining risk stratification [63]. In dMMR CRC, immune profiling further enhances outcome prediction by decoding the tumor immune microenvironment, identifying immunotherapy-responsive subgroups with survival benefits [63]. Our LODDS-based model complements these approaches by quantifying anatomical metastatic spread to lymph nodes. Integrating ctDNA (real-time molecular burden), immune signatures (microenvironmental context), and LODDS (structural progression) offers a multidimensional framework to synergistically optimize prognostic accuracy. For instance, combining ctDNA clearance with immune activation markers could pinpoint durable responders, while LODDS stratifies anatomical risk, enabling tailored therapeutic de-escalation or intensification.

While our study demonstrates that LODDS (log odds of positive lymph nodes) holds promise in refining adjuvant chemotherapy decision-making, several limitations warrant cautious interpretation. First, the increasing adoption of neoadjuvant immunotherapy in dMMR CRC, supported by high pathological response rates in recent trials, may limit the generalizability of our findings to surgically managed cohorts in this evolving therapeutic landscape. Second, our models were derived and internally validated within a single-center retrospective cohort, lacking external validation in diverse populations with detailed dMMR-specific pathological and treatment data, which is critical given known biological and therapeutic heterogeneity across demographics. Third, the omission of postoperative chemotherapy data introduces potential confounding, as adjuvant therapy universally recommended for stage III CRC may independently influence survival outcomes (DFS/OS), thereby biasing the observed prognostic value of LODDS. Furthermore, the retrospective design inherently restricted causal inference due to unmeasured confounders (e.g., treatment adherence, socioeconomic factors) and selection bias from excluded patients with incomplete follow-up. Finally, emerging trends toward lymph node preservation in dMMR CRC (to optimize immunotherapy efficacy) may challenge the clinical utility of LODDS in non-resected populations. Future studies should prioritize integrating LODDS with immunotherapy-specific biomarkers, such as T-cell clonality and ctDNA dynamics, and validate these models in prospective cohorts to address these limitations and advance precision risk-adaptive strategies.

## 5. Conclusions

In non-metastatic dMMR CRC patients, the prognostic value of certain traditional factors was found to be diminished, with pN staging failing to effectively differentiate among pN+ patients. LOODS was identified as a superior independent prognostic factor compared with pN staging and was shown to be effective in further stratifying pN+ patients, influencing both OS and DFS. A prognostic model combining LOODS with age demonstrated superior predictive performance compared with traditional TN staging. LOODS may serve as a valuable tool for guiding postoperative treatment in non-metastatic dMMR CRC patients. LOODS could refine decision-making, potentially sparing select dMMR pN+ patients from unnecessary adjuvant chemotherapy.

## Figures and Tables

**Table 1:** Univariate and multivariate Cox analyses of risk factors for OS.

Variable		Univariate Analysis HR (95% CI)	*p*-Value	Multivariate Analysis HR (95% CI)	*p*-Value
Sex	Female	Reference		NI	
	Male	1.027 (0.596–1.770)	0.923		
Age	LOCRC	Reference		Reference	
	EOCRC	0.452 (0.213–0.959)	0.038	0.394 (0.185–0.839)	0.016
Location	Rectum	Reference		NI	
	Left-sided colon	0.665 (0.262–1.684)	0.389		
	Right-sided colon	0.949 (0.469–1.917)	0.883		
Surgical procedures	Open	Reference		NI	
	Laparoscopic	0.898 (0.475–1.695)	0.739		
Histologic type	Adenocarcinoma	Reference		NI	
	Mucinous/ Signet ring cell	1.689 (0.758–3.766)	0.200		
Differentiation	Poor	Reference		Reference	
	Moderate/well	0.371 (0.207–0.664)	<0.001	0.557 (0.304–1.023)	0.059
Vascular invasion	Negative	Reference		Reference	
	Positive	3.146 (1.794–5.518)	<0.001	1.056 (0.541–2.062)	0.874
Perineural invasion	Negative	Reference		Reference	
	Positive	2.915 (1.581–5.373)	<0.001	1.147 (0.475–2.771)	0.760
T stage	1/2	Reference		Reference	
	3	4.285 (0.965–19.036)	0.056	2.628 (0.568–12.163)	0.217
	4	11.860 (2.651–53.063)	0.001	4.141 (0.827–20.745)	0.084
N stage	0	Reference		Reference	
	1	7.312 (3.777–14.794)	<0.001	5.547 (2.722–11.303)	<0.001
	2	11.282 (5.349–23.794)	<0.001	7.728 (3.348–17.839)	<0.001
TLNs	<12	Reference		NI	
	≥12	0.757 (0.233–2.459)	0.643		

**Table 2:** Univariate and multivariate Cox analyses of risk factors for DFS.

Variable		Univariate Analysis HR (95% CI)	*p*-Value	Multivariate Analysis HR (95% CI)	*p*-Value
Sex	Female	Reference		NI	
	Male	0.897 (0.564–1.429)	0.648		
Age	LOCRC	Reference		NI	
	EOCRC	0.696 (0.399–1.213)	0.201		
Location	Rectum	Reference		NI	
	Left-sided colon	0.754 (0.347–1.636)	0.475		
	Right-sided colon	0.921 (0.506–1.677)	0.788		
Surgical procedures	Open	Reference		NI	
	Laparoscopic	0.995 (0.591–1.675)	0.984		
Histologic type	Adenocarcinoma	Reference		Reference	
	Mucinous/ Signet ring cell	2.145 (1.122–4.097)	0.021	1.531 (0.783–2.995)	0.213
Differentiation	Poor	Reference		Reference	
	Moderate/well	0.389 (0.238–0.637)	<0.001	0.635 (0.375–1.076)	0.091
Vascular invasion	Negative	Reference		Reference	
	Positive	3.288 (2.030–5.326)	<0.001	0.988 (0.541–1.835)	0.969
Perineural invasion	Negative	Reference		Reference	
	Positive	2.859 (1.708–4.788)	<0.001	1.593 (0.705–3.599)	0.263
T stage	1/2	Reference		Reference	
	3	5.105 (1.229–21.211)	0.025	3.104 (0.758–13.545)	0.113
	4	11.593 (2.785–48.260)	<0.001	3.545 (0.763–16.476)	0.106
N stage	0	Reference		Reference	
	1	5.611 (3.255–9.673)	<0.001	4.147 (2.246–7.657)	<0.001
	2	9.180 (4.984–16.908)	<0.001	6.243 (3.021–12.902)	<0.001
TLNs	<12	Reference		NI	
	≥12	0.621 (0.243–1.590)	0.321		

**Table 3:** Multivariate Cox regression analysis including different staging systems.

Staging System		OS		DFS	
		HR (95% CI)	*p*-Value	HR (95% CI)	*p*-Value
pN	pN0	Reference		Reference	
	pN1	5.550 (2.645–11.648)	<0.001	5.773 (23.346–9.963)	<0.001
	pN2	6.836 (2.800–16.688)	<0.001	9.264 (5.030–17.059)	<0.001
NLN	NLN0	Reference		Reference	
	NLN1	2.670 (1.309–5.444)	0.007	1.923 (1.095–3.378)	0.023
	NLN2	6.242 (2.916–13.360)	<0.001	4.622 (2.511–8.510)	<0.001
LNR	LNR0	Reference		Reference	
	LNR1	7.107 (3.757–13.446)	<0.001	5.178 (3.048–8.794)	<0.001
	LNR2	13.082 (6.140–27.870)	<0.001	10.397 (5.547–19.488)	<0.001
LOODS	LOODS0	Reference		Reference	
	LOODS1	3.683 (699–7.984)	<0.001	3.735 (2.019–6.911)	<0.001
	LOODS2	17.432 (8.758–34.699)	<0.001	12.195 (6.875–21.630)	<0.001

## Data Availability

The raw data supporting the conclusions of this article will be made available by the authors without undue reservation.

## Supplementary Materials

The following supporting information can be downloaded at: https://www.mdpi.com/article/10.3390/curroncol32050254/s1, Figure S1: Flow chart of patient selection process; Table S1: Demographic characteristics of patients in this study; Figure S2: The determination of the optimal cut-off values; Figure S3: ROC analysis of four lymph node-based prognostic models.

## Abbreviations

The following abbreviations are used in this manuscript:

AJCC	American Joint Committee on Cancer
AUC	Area under the curve
C-index	Concordance index
CRC	Colorectal cancer
CSCO	Chinese Society of Clinical Oncology
DFS	Disease-free survival
dMMR	Deficient mismatch repair
EOCRC	Early-onset colorectal cancer
ESMO	European Society for Medical Oncology
LNR	Lymph node ratio
LOCRC	Late-onset colorectal cancer
LODDS	Log odds of positive lymph nodes
MMR	Mismatch repair
MSI	Microsatellite instability
NCCN	National Comprehensive Cancer Network
NLNs	Negative lymph nodes
OS	Overall survival
PLNs	Positive lymph nodes
pMMR	proficient mismatch repair
ROC	Receiver operating characteristic curve
TimeROC	Time-dependent receiver operating characteristic curve
TLNs	Total lymph nodes
