# Supplementary material for: Reassessing the Prognostic Value of Lymph Node Metastasis in Deficient Mismatch Repair Colorectal Cancer
Source: Curr Oncol. 2025 Apr 27;32(5):254. doi: 10.3390/curroncol32050254 (PMC12110690; doi:10.3390/curroncol32050254)

Figure S1. Flow chart of patient selection process.

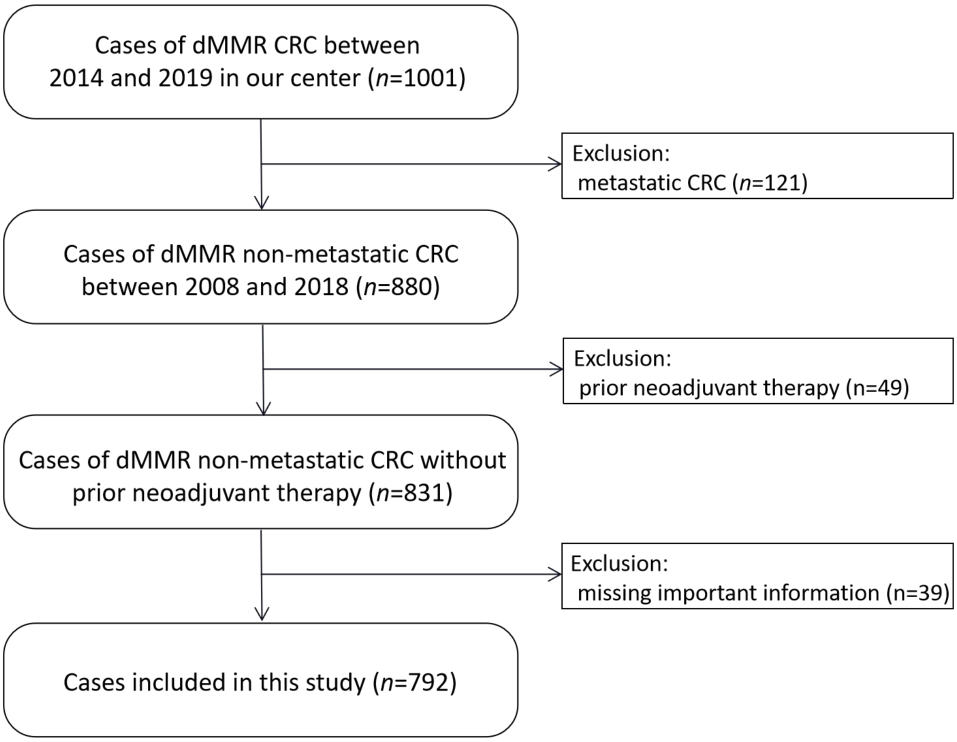

Table S1. Demographical characteristics of patients in this study.

Variable		dmmr CRC (n = 792)
Sex	Male	420 (53.0%)
	Female	372 (47.0%)
Age	LOCRC	576 (72.7%)
	EOCRC	216 (27.3%)
Location	Rectum	130 (16.4%)
	Left-sided colon	150 (18.9%)
	Right-sided colon	512 (64.6%)
Surgical procedures	Open	510 (64.4%)
	Laparoscopic	282 (35.6%)
Histologic type	Adenocarcinoma	657 (83.0%)
	Mucinous/Signet ring cell	68 (8.6%)
	Unknown	67 (8.5%)
Differentiation	Poor	378 (47.7%)
	Moderate/Well	414 (52.3%)
Vascular invasion	Negative	596 (75.3%)
	Positive	196 (24.7%)
Perineural invasion	Negative	656 (82.8%)
	Positive	136 (17.2%)
T stage	1	42 (5.3%)
	2	87 (11.0%)
	3	463 (58.5%)
	4	200 (25.3%)
N stage	0	576 (72.7%)
	1	152 (19.2%)
	2	64 (8.1%)
TLN	< 12	30 (3.8%)
	≥ 12	792 (96.2%)

LOCRC：Late-Onset Colorectal Cancer；EOCRC：Early-Onset Colorectal Cancer

Figure S2. The determination of the optimal cut-off values. Selecting the optimal cut-off values for NLN (**A**), NLR (**C**), and LODDS (**E**) using X-Tile; Grouping NLN (**B**), NLR (**D**), and LODDS (**F**) based on optimal cut-off values.

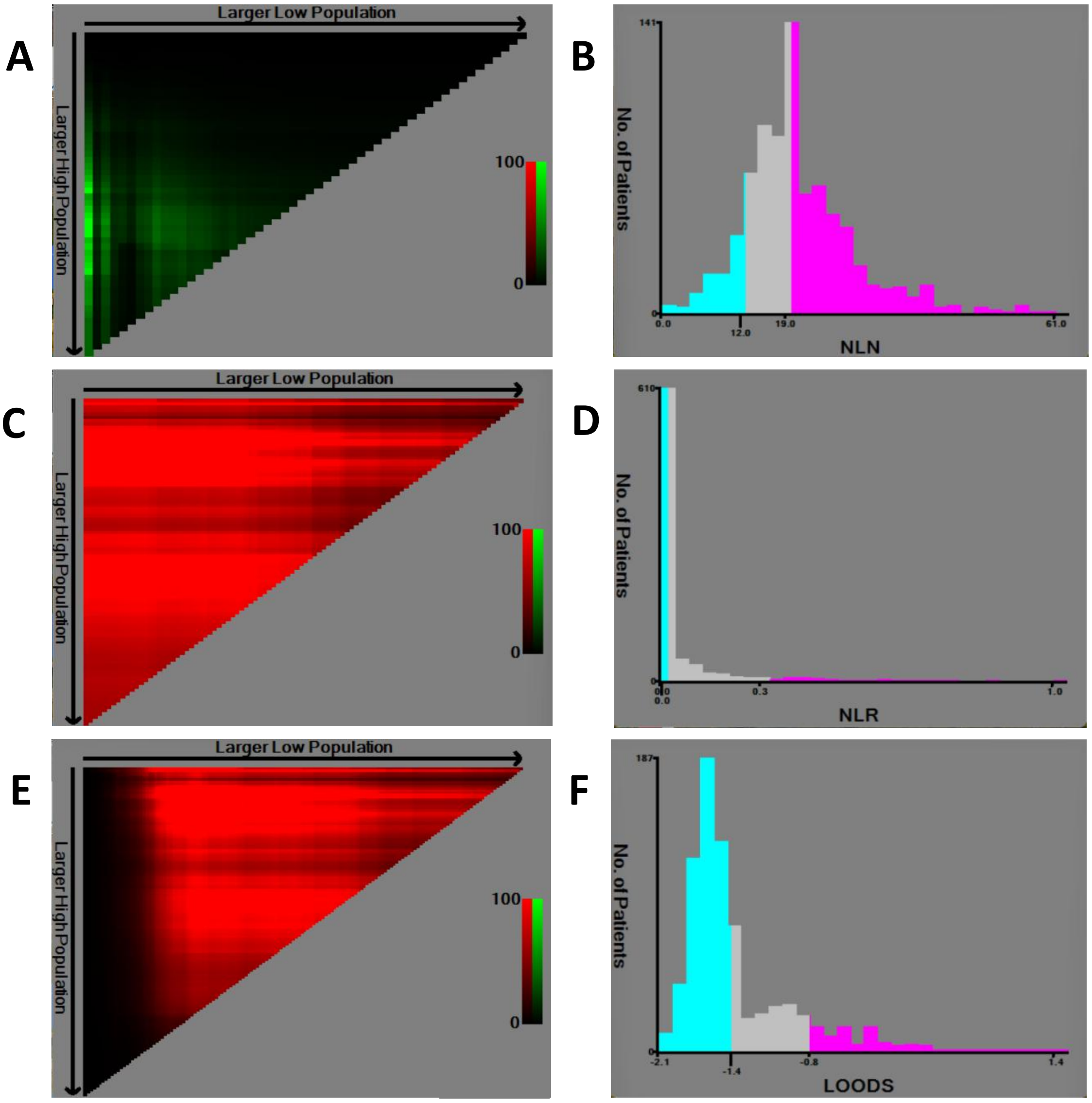

Figure S3. ROC analysis of four lymph node-based prognostic models. ROC curves for OS at (**A**) 1, (**C**) 3, and (**E**) 5 years; ROC curves for DFS at (**B**) 1, (**D**) 3, and (**F**) 5 years.

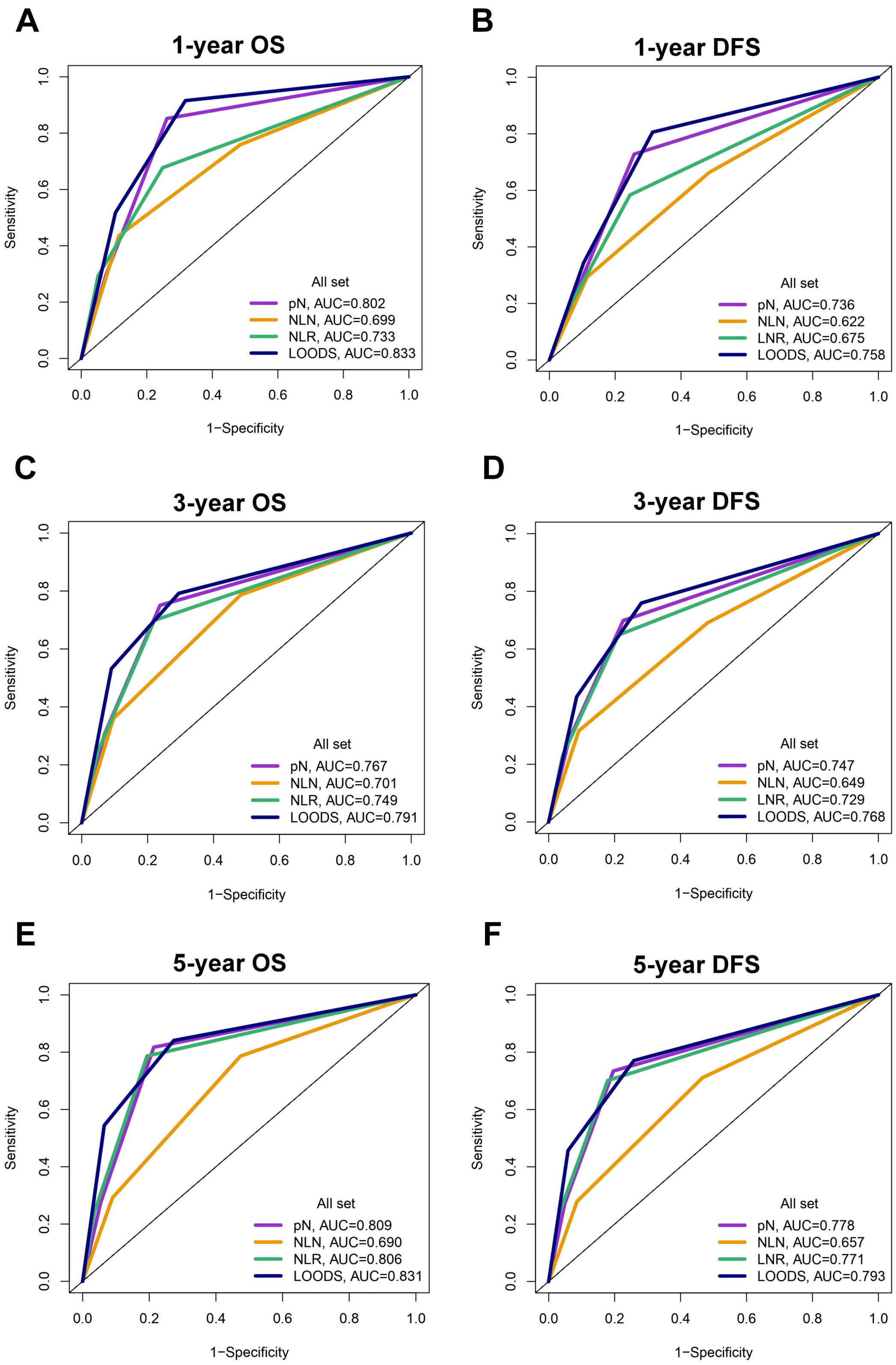

Supplement: Supplementary file 1 [file curroncol-32-00254-s001.zip › curroncol-3546799-supplementary.pdf]
